# The Utility of a Virtual Emergency Medicine Elective for Visiting Medical Students

**DOI:** 10.7759/cureus.43686

**Published:** 2023-08-18

**Authors:** David Chu, Kiran Pandit, Robert Giles, Erica Olsen, Alexander Fortenko, Peter Greenwald, Tiffany Murano, Kaushal Shah, Sophia Lin

**Affiliations:** 1 Emergency Medicine, Massachusetts General Hospital, Boston, USA; 2 Emergency Medicine, Albert Einstein College of Medicine, New York, USA; 3 Emergency Medicine, Memorial Satilla Health, Waycross, USA; 4 Emergency Medicine, Columbia University College of Physicians and Surgeons, New York, USA; 5 Emergency Medicine, Weill Cornell, New York, USA

**Keywords:** educational culture, virtual course, curriculum, medical education, emergency medicine

## Abstract

Background

Away rotations allow emergency medicine (EM)-bound fourth-year medical students to experience a residency program’s educational culture and influence the ranking of residency programs. The financial cost and geographic distance have limited student participation in away electives. In recent years, COVID-19 pandemic-related restrictions on away rotations resulted in the creation of multiple virtual courses. Despite the lifting of restrictions, these courses may still have utility in helping students circumvent barriers to away rotations. Limitations of previously described courses include insufficient student-faculty interaction, which influences students’ understanding of the educational environment. We sought to develop and evaluate a virtual EM elective for fourth-year medical students, focused on student-faculty interaction including precepted patient contact.

Methodology

We developed a two-week virtual EM elective for fourth-year medical students incorporating teaching sessions designed to optimize student-faculty interactions and attending-supervised telemedicine visits. After completion of the course, students completed an anonymous course evaluation.

Results

Course evaluations showed that the course improved students’ understanding of our residency’s educational environment by providing students with access to our residency program. The most frequently cited factors preventing participation in a traditional away elective were financial cost, limit in the allowed number of away rotations, and challenges in finding housing.

Conclusions

We believe this course may be an effective way of improving visiting students’ understanding of the educational culture of our EM residency program. Thus, although pandemic-related restrictions have been lifted, this course may serve as a valuable adjunct to the traditional away EM rotation.

## Introduction

One reason medical students pursue “away” rotations (i.e., rotations at sites that are not part of the student’s institution) is to better understand a residency program’s educational culture [1]. This understanding contributes to a student’s appreciation of “fit” (how well the residency program matches the student’s individual needs and personal goals), arguably the most important factor for students in ranking residency programs [2]. In May 2020, in response to the COVID-19 pandemic, the Coalition for Physician Accountability (COPA) discouraged visiting rotations for 2020-2021 [3]. In 2021, COPA recommended limiting fourth-year students to one away rotation for 2021-2022 [4]. Due to these restrictions, medical schools created virtual electives across multiple specialties [5-9].

Virtual EM rotations, lasting one to four weeks, have been described [10-14]. Approaches utilized in these courses to facilitate active learning include faculty-led small group sessions [13] and case-based teaching sessions [12,14]. One rotation incorporated asynchronous case-based discussions to increase faculty-student interactions [12]. In another course, students participated in faculty-supervised telemedicine patient encounters [11], but all encounters were follow-up visits after an in-person emergency department visit. However, as with virtual courses in other specialties, most EM courses had limited opportunities for students to interact with faculty and engage in faculty-precepted patient care [5,10,15-18].

COPA lifted restrictions on visiting electives for the 2022-2023 academic year [19]. Despite this, virtual away electives may continue to serve an important role in enabling students to access and experience residency programs outside their home institutions. Pre-pandemic, financial costs and geographic distance were the most often reported limitations to away rotations [20-24]. In one study, 35% of medical students could not complete an away rotation because of cost [25]. Time commitment, family responsibilities, and elective availability are other barriers [1,23,24,26,27].

During the COVID-19 pandemic, we faced the need for a new virtual EM elective. The literature revealed a gap in describing the curriculum that, in addition to delivering outstanding educational value, would also best deliver meaningful student-faculty interaction. This interaction is important for students’ understanding of our residency program’s educational culture, which, in turn, influences their ranking of programs.

## Materials and methods

Development process

In designing this course, we sought to maximize interactions between students and faculty as the basis for delivering outstanding educational value. We incorporated advanced EM content appropriate for students who had already completed a four-week EM clerkship. The three primary elements of the course were (1) synchronous educational sessions, (2) asynchronous online discussion boards, and (3) virtual urgent care telemedicine shifts. We utilized situated cognition, case-based learning, and cooperative learning principles in designing the course and requested that faculty utilize these principles while teaching. We omitted the teaching of invasive procedures as we felt these are best learned in person.

Synchronous components of the course were conducted using Zoom (San Jose, CA, USA), and included six case-based discussions, two electrocardiogram (ECG) sessions, and two simulations. Senior residents with attending preceptors led two case-based sessions, showcasing residents as educators in our residency program. Faculty led all other synchronous sessions. We divided students into groups of four to five to optimize student-faculty interactions. Students also participated in the residency program’s morning report series and weekly conference, as well as the department’s monthly ultrasound and toxicology conferences.

Students participated in asynchronous online discussion boards of their choice via Slack (San Francisco, CA, USA). These discussion boards were each centered on a subspecialty; options included Pediatric EM, Geriatric EM, Emergency Ultrasound, Austere Medicine, and Toxicology. Discussions featured challenging cases, controversial topics, and high-impact articles with at least two specialty faculty to promote robust discussions. We encouraged faculty and students to participate in discussions daily.

We assigned each student two four-hour telemedicine shifts during which they participated in the care of virtual urgent care patients. Students viewed online telemedicine education modules in preparation. On shift, students interviewed and examined patients under direct faculty supervision. The faculty then completed the patient encounter and debriefed the case with the student. Students met preceptors on WebEx (San Jose, CA, USA) and conducted patient visits via Zoom.

Students completed an anonymous course evaluation inquiring about their satisfaction with curricular elements, understanding of the residency’s educational culture, accessibility of the elective, and barriers to pursuing an in-person away rotation. The Weill Cornell Medicine institutional review board (IRB) determined this project to be a quality improvement initiative, exempting it from IRB approval.

The implementation phase

We advertised the elective through the Visiting Student Learning Opportunities (VSLO) portal, Society for Academic Emergency Medicine Residents and Medical Students listserv, and institutional social media accounts. Completion of a four-week in-person EM clerkship or sub-internship was a prerequisite for enrollment. Nine students enrolled in the course in October 2021. They participated in a full-time curriculum of didactics, telemedicine shifts, and asynchronous coursework. Students could opt for additional telemedicine shifts.

The elective was a pass/fail course based on attendance and participation, as course directors felt tiered grades could not be given for an EM course without in-person interactions with emergency department patients.

Several students missed course content to attend residency interviews and made up missed coursework by reviewing recordings of missed sessions or reading relevant material. Each student missed no more than one session of each type of educational activity; no students missed telemedicine shifts as these were scheduled around student availability. Telemedicine faculty provided students with real-time feedback during shifts and completed student assessments after each shift. Students completed faculty assessments and a course evaluation at the end of the course.

## Results

Our primary outcome was student ratings of the course’s effectiveness in deepening their understanding of our residency’s educational culture. A secondary outcome was student ratings of the course’s effectiveness in addressing barriers to participation in an in-person away rotation.

Eight of nine students completed the course evaluation. Seven (88%) were “extremely satisfied” with the course, while one (13%) was “somewhat satisfied” (Table 1).

**Table 1:** Evaluation question #1.

Evaluation question: How satisfied are you with this course overall?	Student responses (n = 8)
Extremely satisfied	7 (88%)
Somewhat satisfied	1 (13%)
Neither satisfied nor dissatisfied	0 (0%)
Somewhat dissatisfied	0 (0%)
Extremely dissatisfied	0 (0%)

All eight students indicated the course improved their understanding of the residency’s educational culture “extremely well” or “very well” (Table 2).

**Table 2:** Evaluation question #2.

Evaluation question: How well did this elective improve your understanding of our EM residency culture and educational environment?	Student responses (n = 8)
Extremely well	5 (63%)
Very well	3 (38%)
Moderately well	0 (0%)
Slightly well	0 (0%)
Not well at all	0 (0%)

Six (75%) indicated that the virtual nature of this course allowed them access to our residency that they would not have had otherwise (Table 3).

**Table 3:** Evaluation question #3.

Evaluation question: Did the virtual nature of this course allow you access to our EM residency that you would not have had otherwise?	Student responses (n = 8)
Yes	6 (75%)
No	2 (25%)

The most frequently cited barriers to an in-person away rotation were the cost of an away rotation (5/8, 63%), the limit in the number of allowed away rotations (5/8, 63%), and the challenge of finding housing (4/8, 50%). Other barriers were the time required to travel to an away rotation (2/8, 25%) and family/personal obligations (1/8, 13%) (Table 4).

**Table 4:** Evaluation question #4.

Evaluation question: Please check if any of the following are barriers for you in participating in away rotations (check all that apply)	Student responses (n = 8)
Cost of an in-person away rotation	5 (63%)
Limit in number of away rotations allowed by my medical school	5 (63%)
Challenge of finding housing	4 (50%)
Time required to travel to an away rotation	2 (25%)
Family/personal commitments or obligations	1 (13%)

Seven respondents applied to EM residency. All seven indicated that the elective increased the likelihood they would rank our residency among their top 10 programs. One student successfully matched into our residency.

Excerpts from the free-text portion of the anonymous course evaluations demonstrate positive student feedback:

“The case discussions and sim sessions were really helpful because we were in small groups and given a lot of interaction and attention by faculty - kept us more engaged and learned more.”

“Subspecialty tracks were a fantastic way to deep dive into a sub-topic in EM with faculty experts in that field, this was an extremely unique opportunity that I haven't had elsewhere.”

“Really enjoyed my urgent care shifts … [I received] great constructive feedback on my interactions with patients.”

## Discussion

This course improved students’ understanding of our residency’s educational culture and increased access to our residency program. Students were highly satisfied with this course. We attribute this success to the design of the elective which maximized opportunities for meaningful, active student-faculty interactions. Small-group teaching and the incorporation of asynchronous specialty tracks and faculty-precepted telehealth facilitated these interactions.

Other institutions can readily replicate most components of this course. As a result of the COVID-19 pandemic, many residency programs continue to conduct at least a portion of their educational events over Zoom, and faculty have developed skills in using online platforms for teaching. Many curricular elements did not require additional faculty time, as students were joining sessions that were already occurring for other audiences. This includes the weekly residency conference, morning report conferences, ultrasound tape conference, and toxicology conference. Faculty engagement was requested for two simulation sessions, two ECG sessions, and six case discussions, totaling 10 hours of faculty time. Two case discussions were led by residents, and other institutions may wish to have more of these sessions led by residents, rather than faculty if faculty time is a resource limitation. Faculty engagement in the Slack discussion boards was asynchronous; faculty controlled the timing of their engagement, and we estimate that each faculty member spent around two to three hours on this over the course.

The telehealth shifts provided a unique opportunity for student-faculty interaction with live patients, despite the virtual nature of the course. During these shifts, faculty were able to directly observe students’ patient care skills, function as role models for students, and provided real-time feedback to students immediately after every patient encounter. Although not all healthcare institutions have the infrastructure to incorporate telemedicine shifts into a virtual course, the number of institutions offering telehealth services has dramatically increased since the pandemic [28] and continues to grow.

A major limitation in determining the impact of this innovation is our small sample size, which limited our ability to assess the effectiveness of our new curriculum. Higher enrollment would have allowed for a more robust assessment of EM knowledge and skills; gains in these areas were clearly reflected in the students’ comments but were not objectively measured in this study. Higher enrollment would have also allowed for analysis of the educational effectiveness of individual course elements. We have, however, successfully demonstrated proof of concept. Earlier and more widespread advertisements of this course would have increased enrolment and allowed for a stronger assessment of educational value.

With future iterations of this course, the next steps may include studying the perspectives of faculty and residency leadership regarding the effectiveness of the elective in increasing familiarity with potential residency applicants. Additionally, determining the impact of participation in this course on both student and program rank lists and match results can also be examined.

With the lifting of restrictions on medical student away rotations, student satisfaction with this course may change, as in-person away rotations were not available at the time of this course. Despite this, our course still has utility primarily because the virtual aspect may reduce barriers that impede students from participating in traditional away rotations. Our students listed financial constraints, time constraints, logistical challenges, and family obligations as barriers. Virtual electives would allow students to circumvent most of these barriers.

## Conclusions

The COVID-19 pandemic pushed medical educators to go beyond traditional methods of delivering education. Virtual rotation development was a resulting innovation. Even after the lifting of pandemic-related restrictions, virtual courses can serve an important role in increasing medical student access to other institutions. This access is important for students seeking postgraduate training as it allows them to better understand a residency’s educational environment. Thus, we believe a virtual elective may be a valuable permanent adjunct to traditional away rotations.
